# VALIDATING SILICONE WRISTBANDS TO MEASURE PESTICIDE EXPOSURES AMONG OLDER ADULTS -- PROOF-OF-CONCEPT STUDY

**DOI:** 10.1093/geroni/igz038.3298

**Published:** 2019-11-08

**Authors:** Marianne Chanti-Ketterl, Brenda L Plassman, Christine Parks, Nicholas Herkert, Julie Fleenor, Sharon Zhang, Heather Stapleton

**Affiliations:** 1 Duke University, Durham, North Carolina, United States; 2 Epidemiology Branch, National Institute of Environmental Health Sciences, Research Triangle Park, North Carolina, United States; 3 Nicholas School of the Environment, Duke University, Durham, North Carolina, United States; 4 Department of Psychiatry and Behavioral Sciences, School of Medicine, Duke University, Durham, North Carolina, United States

## Abstract

Silicone wristbands have been used to measure exposure to pesticides and other chemicals among children and younger farm workers, but not in older adults. Thus, we aimed to examine exposure to pesticides using silicone wristbands in a small cohort of older adults living on agricultural land, with variable contact with fields and pesticides. We also investigated correlations between pesticide levels on wristbands and urinary pesticide metabolites. Organophosphate (OPH) pesticides and several organochlorines were measured in wristbands worn by 15 males age 70+ (10 farmers using pesticides and 5 non-farmers with no recent pesticide use). Wristbands were worn continuously for 5-days. End-of-day urine samples were collected on days 1-3-5. Spearman correlations and Wilcoxon Scores were calculated. Five pesticides were quantified in the wristbands and detection frequencies ranged from 40-90%. In urine,12 OPH metabolites were quantified, but only 5 were detected in >50% of the samples. None of 5 urinary herbicides were detected. Imputation was performed by dividing minimum-detect by square-root-2. Malathion was only detected in farmers compared to non-farmers. Correlations between OPH urinary metabolites and wristband were examined but only two were significant and were negative in direction. Notably, organochlorine DDE on the wristbands was significantly correlated with 3 OPH metabolites. These unexpected relationships, based on small numbers, suggest a need to replicate this work in a larger study sample to explore potential for confounding or mixtures in future studies of pesticides and health in older farmers.

